# Winning during a pandemic: epidemiology of SARS-CoV-2 during EURO2020 in Italy

**DOI:** 10.1017/S0950268822000723

**Published:** 2022-04-22

**Authors:** Flavia Riccardo, Emanuela Maria Frisicale, Giorgio Guzzetta, Federica Ferraro, Stefano Merler, Guido Maringhini, Matteo Spuri, Daniele Petrone, Maria Cristina Rota, Alessia Rapiti, Ulrico Angeloni, Pasqualino Rossi, Marco Tallon, Stefania Giannitelli, Patrizio Pezzotti, Martina Del Manso, Antonino Bella, Francesco Paolo Maraglino

**Affiliations:** 1 Department of Infectious Diseases, Istituto Superiore di Sanità, Rome, Italy; 2 Ministry of Health, Directorate General of Health Prevention, Rome, Italy; 3 Fondazione Bruno Kessler, Trento, Italy; 4 Ministry of Health, Directorate General for Communication and European and International Relations, Rome, Italy

**Keywords:** COVID-19, infectious disease epidemiology, outbreaks, transmission, surveillance system

## Abstract

**Introduction:**

EURO2020 generated a growing media and population interest across the month period, that peaked with large spontaneous celebrations across the country upon winning the tournament.

**Methods:**

We retrospectively analysed data from the national surveillance system (indicator-based) and from event-based surveillance to assess how the epidemiology of severe acute respiratory syndrome coronavirus 2 (SARS CoV-2) changed in June–July 2021 and to describe cases and clusters linked with EURO2020.

**Results:**

Widespread increases in transmission and case numbers, mainly among younger males, were documented in Italy, none were linked with stadium attendance. Vaccination coverage against SARS-CoV-2 was longer among cases linked to EURO2020 than among the general population.

**Conclusions:**

Transmission increased across the country, mainly due to gatherings outside the stadium, where, conversely, strict infection control measures were enforced. These informal ‘side’ gatherings were dispersed across the entire country and difficult to control. Targeted communication and control strategies to limit the impact of informal gatherings occurring outside official sites of mass gathering events should be further developed.

## Introduction

A mass gathering event (MGE) is defined as a gathering of 1000 persons or more at a specific location for a specific purpose for a defined period of time [1]. These events strain national planning and response resources and are linked with increased infectious disease transmission risk [2, 3].

However, improvement in planning, infrastructures and prevention and control measures have in recent years allowed many MGEs to be held safely, also during Public Health Emergencies of International Concern [4]. The planning and implementing complexities of MGEs have notably increased during the coronavirus disease 2019 (COVID-19) pandemic [5, 6].

The European Soccer Championship (EURO) is a global sport event. EURO2020, postponed to 2021, exceptionally took place in 11 different host countries opening in Rome (11 June 2021) and closing in London (11 July 2021). The event engaged 24 National teams in 51 live matches allowing spectators under diverse COVID-19 restrictions with a cumulative audience exceeding five billion people [7]. The Italian national team played seven matches, the first three (11, 16 and 20 June 2021) were played in the *Olimpico Stadium* in Rome, the remaining ones in London (26 June, 6 and 11 July 2021) and in Munich (2 July 2021) [8]. The *Olimpico Stadium* also hosted an additional match, Ukraine *vs*. England, on 3 July 2021.

## Methods

Since February 2020, Italy notifies all laboratory-confirmed SARS-CoV-2 human infections to a national case-based surveillance system (hereby indicator-based surveillance) as previously described [9]. We analysed the epidemiology of SARS-CoV-2 in Italy before, after and during the EURO2020 MGEs by defining 1 June – 31 July 2021 as the observation period of this study. We extracted data, consolidated as of 11 November 2021, on all cases of laboratory-confirmed SARS-CoV-2 infection diagnosed in the observation period summarising it by date of symptoms' onset and of diagnosis. We estimated the net reproduction number (Rt) as previously described [9] at national and Region/Autonomous Province (AP) level, putting this in relation to dates of relevant EURO2020 events. We summarised data of all confirmed cases by age and sex and compared this distribution in time intervals of 15 days.

### Retrospective analysis of cases/clusters with exposures linked with EURO2020 MG events

#### Indicator-based surveillance

The Italian surveillance system includes, for each notified case, two free text variables in which the most likely place of exposure, when known, and any additional annotations are reported. We performed a key word search on those two variables among all cases diagnosed in the observation period using the following search terms: euro, final*, calcio, stadio, Olimpico, partit*, 2020, semifinal*, festeggiament*, Italia, max*, piazza, which were the Italian words for euro, final, football, stadium, Olimpico (the name of the stadium), match, 2020, semifinal, celebrations, Italy, big (as in big screen), square.

The asterisk was used to include all possible declinations of an individual search term.

As search terms were not all EURO2020-specific, all cases identified (hereby cases linked to EURO2020) in this way were also manually checked to exclude those unrelated to EURO2020. We performed a descriptive analysis on the remaining subset of cases and aggregated data by Region/AP of diagnosis.

To assess vaccination status at the time of diagnosis (or at the time of symptoms' onset if the date of diagnosis was unavailable), we linked all cases linked with EURO2020 to the national vaccination register. We defined cases as ‘unvaccinated’ if they had never received a vaccine dose or had received a first dose less than 15 days before, ‘vaccinated – partial primary cycle’ if they had received only one dose of a two doses vaccination regimen or a second dose less than 15 days before, and ‘vaccinated – full primary cycle’ if they had completed primary vaccination (single or two doses regimen) at least 15 days before. We compared the vaccination coverage of SARS-CoV-2 linked to EURO2020 with the vaccination coverage documented in the general Italian population (all age groups) as of 30 June 2021 (mid-date in the observation period).

#### Event-based surveillance

Event-based surveillance is performed in Italy by the Italian Network of Epidemic Intelligence [3]. In order to complement the analyses described, a key word search, restricted to the observation period, was performed in Italian on *Googlenews* using the search terms ‘*focolaio*’ or ‘*cluster*’ in combination with at least one of the following search terms: *euro, semifinale, finale, partita, calcio, stadio, Olimpico, festeggiamenti, Italia, maxischermo, festa and nazionale.* We defined an event as a cluster of severe acute respiratory syndrome coronavirus 2 (SARS-Cov-2) infections associated with EURO2020 MG events in Italy. Each signal was assessed by an event-based surveillance analyst who selected the events, listing them by setting and Region/AP of occurrence.

## Results

**Fig. 1.**
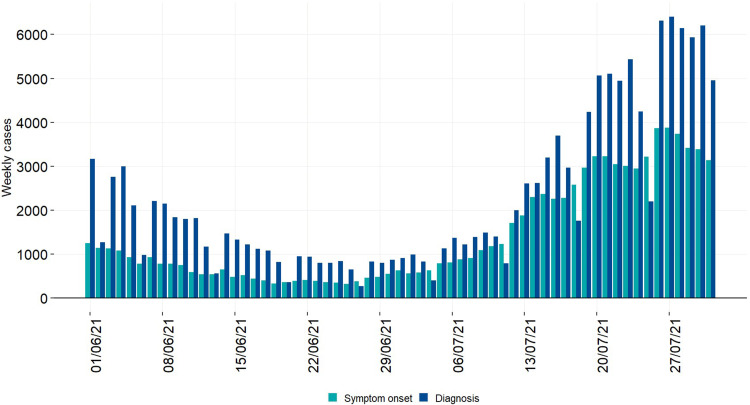
Newly diagnosed cases of SARS-CoV-2 infection (*n* = 137.985), by date of symptoms' onset (green) and date of diagnosis (blue), Italy, 1 June – 31 July 2021 (data consolidated as of 11 November 2021).

**Fig. 2.**
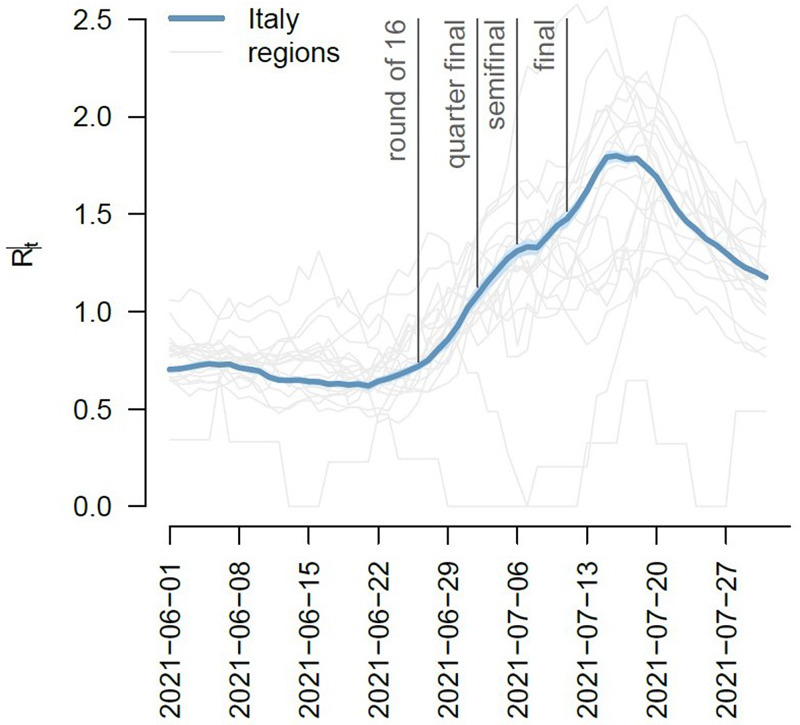
Net reproduction number (Rt) at national (thick line) and regional (thin line) level by week in relation with main EURO2020 MG events, Italy, 1 June – 31 July 2021 (data consolidated as of 11 November 2021).

**Fig. 3.**
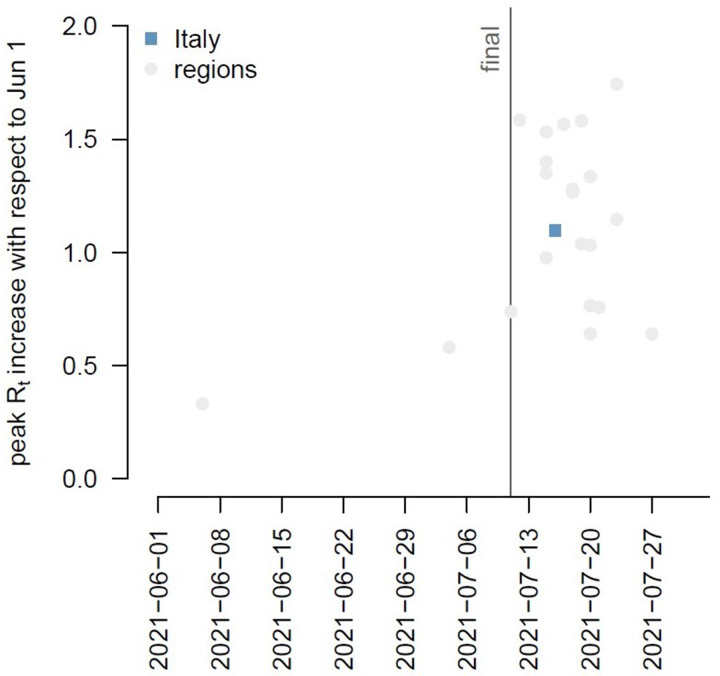
Peak increase in the net reproduction number (Rt) at national (blue square) and regional (grey squares) level compared with 1 June 2021 in relation with the EURO2020 final match, Italy, 1 June – 31 July 2021 (data consolidated as of 11 November 2021).

**Fig. 4.**
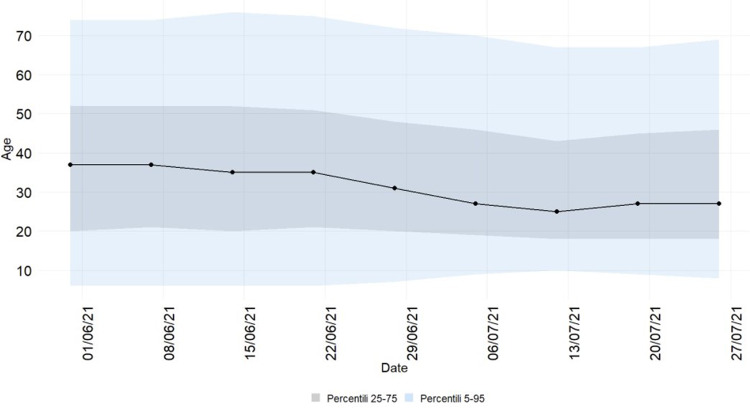
Median age of newly diagnosed cases of SARS-CoV-2 infection and percentile ranges (25–75 and 5–95), Italy, 1 June – 31 July 2021 (data consolidated as of 11 November 2021).

**Fig. 5.**
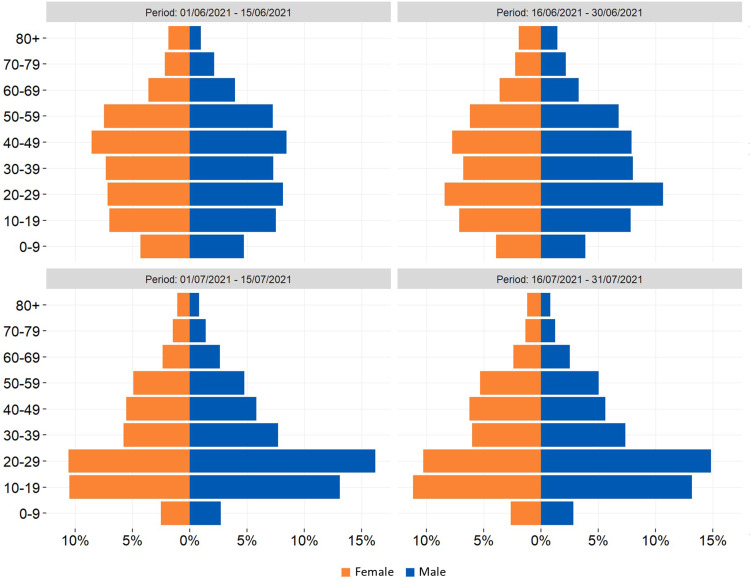
Fifteen-day distribution of newly diagnosed cases of SARS-CoV-2 infection (*n* = 137.985), age group and sex, Italy, 1 June – 31 July 2021 (data consolidated as of 11 November 2021).

Between 1 June and 31 July 2021, 137.993 confirmed cases of SARS-CoV-2 infections (daily range: 274–6404; 52.9% Male; 79.8% locally acquired) were diagnosed in Italy. We observed an increasing trend in the number of new infections with a peak on 27 July 2021 (Fig. 1). The Rt nationally crossed the epidemic threshold in the beginning of July and continued increasing until 5 days after the final EURO2020 match (Fig. 2). The greatest Rt increase, compared with 1 June 2021, occurred between 1 and 14 days after the final match in 80% of all Italian Regions/APs (17/21) (Fig. 3). The median age of cases in the observation period was 29 years (IQ range 24–39) with a decreasing trend between 20 June and 20 July 2021 (Fig. 4). By comparing the age and sex pyramids of cases in 15-day intervals (Fig. 5), we observed a predominance of newly diagnosed SARS-CoV-2 cases among males aged between 10 and 29 years in July 2021.

### Retrospective analysis of a subset of cases linked with EURO2020

#### Indicator-based surveillance

**Fig. 6.**
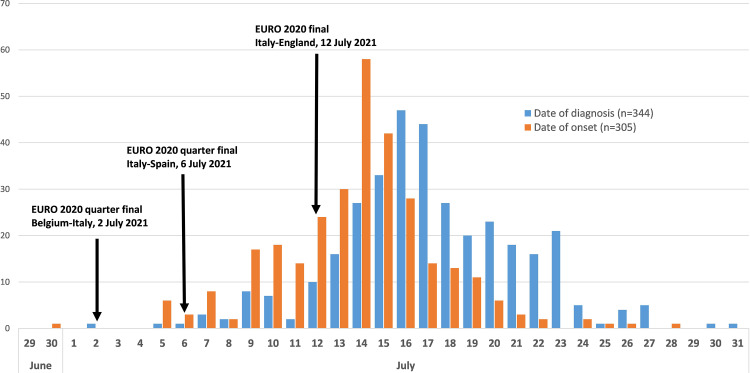
Newly diagnosed cases of SARS-CoV-2 infection with reported most likely exposure linked with EURO2020 MG events (*n* = 344), Italy, 1 June – 31 July 2021 (data consolidated as of 11 November 2021).

**Fig. 7.**
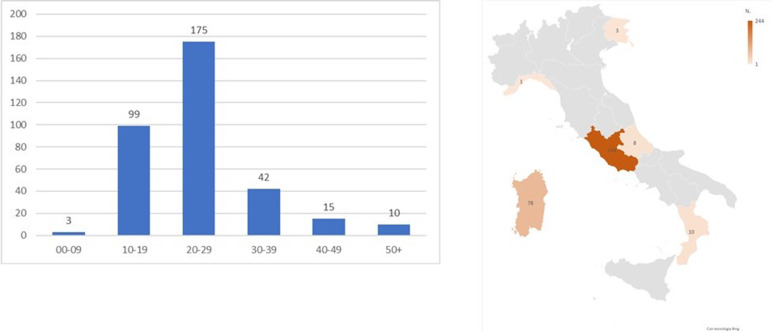
Newly diagnosed cases of SARS-CoV-2 infection with reported most likely exposure linked with EURO2020 MG events (*n* = 344), by age group (left) and Region/AP of diagnoses (right), Italy, 1 June – 31 July 2021 (data consolidated as of 11 November 2021).

In the observation period, we identified 344 new cases of SARS-CoV-2 infection (77% male, median age 21 years, 96.5% locally acquired) with reported exposures linked with EURO2020 MG events. Most cases developed symptoms after the EURO2020 final match (Fig. 6) with a peak on the 14th of July 2021. In all cases, the exposure was linked to victory parties outside stadiums. As shown in Figure 7, most cases were observed in the 20–29 age group (*n* = 175, 51%) and diagnosed in the Lazio Region (*n* = 244, 71%). All other cases were diagnosed across the country with no evident distribution gradient.

Most cases were unvaccinated (*n* = 264; 76.74%), 51 had partially completed the primary vaccination cycle (14.83%) and only 29 cases (8.43%) were fully vaccinated. Compared with the general population, we observed that the cases linked with EURO2020 were more frequently unvaccinated or partially vaccinated (Table 1).

**Table 1.** Vaccination status of cases linked to EURO2020 at the time of diagnosis/onset of symptoms and of the general population (all age groups) as of 30 June 2021, Italy

Vaccination status	Cases linked to EURO2020 (all age groups), at time of diagnosis/symptoms' onset		General population (all age groups), Italy, 30 June 2021	
	*N*	%	*N*	%
Unvaccinated	264	76.74	30.512.808	51.49
Vaccinated – partial primary cycle	51	14.83	13.162.072	22.21
Vaccinated – full primary cycle	29	8.43	15.582.686	26.30
Total	344		59.257.566	

#### Event-based surveillance

We identified six different events occurring in four different Regions with reported clusters ranging from 3 to over 100 cases (Table 2). All events reported were linked to aggregations either in private settings, pubs/other public buildings, or public squares. The largest cluster, reported several times, was associated to a single gathering in a pub in Rome during the Italy-Belgium match (2 July 2021).

**Table 2.** News items reporting clusters of SARS-CoV-2 infections associated with the EURO2020 MGs in Italy identified through event-based surveillance, Italy, 1 June – 31 July 2021

ID	Region/Autonomous province	Number of cases associated with the cluster	Transmission setting described	Title of the article (Italian)	Reference	Date of publication
1	Veneto	6	Multiple aggregation events including the viewing of the Italy-Austria match on a public big-screen	Focolaio Covid nel Feltrino, tutti ventenni (il compleanno, la partita dell'Italia, la birreria…)	[10]	02/07/2021
2	Toscana	11	Cluster following big-screen viewing of the Italy-Belgium match in Florence	Firenze, effetto Europei: focolaio al maxischermo	[11]	17/07/2021
3	Umbria	Not specified	Celebration in a public square following the EURO2020 final match	Covid in Umbria, casi raddoppiati per il focolaio degli Europei. Vaccino completo a metà popolazione	[12]	21/07/2021
4	Lazio	>90	Cluster associated with viewing the Italy-Belgium match in a pub in Rome (Monteverde)	Variante Delta, nuovi focolai in Italia: da Roma a Pantelleria	[13]	19/07/2021
4	Toscana	At least 3	Cluster associated with viewing the final EURO 2020 match in an open air establishment in Florence	Variante Delta, nuovi focolai in Italia: da Roma a Pantelleria	[13]	19/07/2021
5	Lazio	>70	Cluster associated with viewing the Italy-Belgium match in a pub in Rome (Monteverde)	Covid, la variante ‘Europeo’: focolaio a Roma per Italia-Belgio	[14]	16/07/2021
6	Lazio	>100	Cluster associated with viewing the Italy-Belgium match in a pub in Rome (Monteverde)- updated	Covid, cluster a Ostia: 26 contagiati durante gli Europei. A Ciampino focolaio in un oratorio	[15]	21/07/2021
6	Lazio	26	Cluster associated with viewing UEFA 2020 football matches in an establishment in Rome (Ostia)	Covid, cluster a Ostia: 26 contagiati durante gli Europei. A Ciampino focolaio in un oratorio	[15]	21/07/2021

## Discussion

EURO2020 generated an increasing interest in Italy, culminating in large spontaneous celebrations across the country after the national team won the final match. Informal celebrations became more and more common across the country until 11 July 2021, when a ‘frenzy night’ of celebrations took place in many Italian cities [16]. The change in behaviour was widespread enough to determine a rapid and almost synchronous increase in transmission across the country with the highest peaks in the two weeks following the final match. After this time, transmission decreased and then remained below the epidemic threshold until the fall [17]. Consistently, we observed an increase in the number of new cases of SARS-CoV-2 infection in July 2021, mainly among younger males that usually hold a greater interest in this type of event. These observations are in line with a previous report from a different European country [18].

Most of the cases reported in the surveillance system with a link to EURO2020 MG events were reported in the Lazio Region where the *Olimpico* stadium is located. However, our data do not support the hypothesis that the stadium itself was a transmission setting. Firstly, transmission increased consistently across most of the country at the same time. Secondly, we could not observe any relationship of proximity between the number of SARS-CoV-2 infections linked to EURO2020 and the location of the *s*tadium. Thirdly, none of the EURO2020 linked cases and clusters were directly or indirectly associated with stadium attendance.

Several factors could have contributed to limiting transmission inside the stadium. Firstly, ongoing COVID-19 restrictions including indoor or outdoor mask wearing and physical distancing [19, 20] were in force. Secondly, a strict COVID-19 security protocol at the stadium itself during the championship, that limited live attendance at 25% of total stadium capacity, established access verification and managed spectator flows. All spectators older than six accessed the stadium only if they exhibited proof of at least one of the following: negative SARS-CoV-2 test performed within the previous 48 hours, vaccination (at least one dose), prior SARS-CoV-2 infection ended within the previous 6 months. Cross flows and overcrowding at the entrance were limited by assigning spectators in advance specific entrances with travel directions [21].

The increase in the cases in Italy was driven by locally acquired infections, and the percentage of autochthonous cases among those retrospectively linked to EURO2020 was even higher than the national average. This could be in part due to strict cross-border requirements that included showing a negative swab-test, a vaccination/recovery certificate before arrival or quarantining upon arrival and testing again at the end of the quarantine period [22, 23]. Restrictions also limited access to national EURO2020 delegations, with only limited exemptions.

The cases and clusters we were able to link retrospectively with EURO2020 were associated with aggregations in private settings, in pubs/other public buildings, or in public squares. These settings are dispersed and more difficult to control. The extent of transmission could be explained by the combination of behaviour change (increased interpersonal contacts, poor observation of physical distancing and mask wearing) and the concurrent emergence of the more transmissible Delta SARS-CoV-2 variant [24].

Almost all the cases linked to the EURO2020 event were either unvaccinated or partially vaccinated according to the primary vaccination schedule that in Italy includes either two doses of an mRNA vaccine (BNT162b2 or mRNA-1273), a single dose of Ad26.COV.S (Johnson & Johnson) or a dose of ChAdOx1 nCoV-19 followed either by a second dose of a homologous or heterologous (mRNA) vaccine.

Vaccination coverage in this group was lower compared with the national vaccination coverage registered in the general population at the time, possibly suggesting a lower SARS-CoV-2 risk perception in this population.

As no specific additional surveillance was set-up nationally to monitor this MGE, the study is limited by the kind of analysis that we could perform: i.e. a comprehensive retrospective analysis of multiple routine surveillance systems (indicator and event-based). For this reason, the number of cases and clusters linked to EURO2020 is limited to those detected and reported and may be substantially underestimated. We did not identify any case of infection who had a history of attending the stadium, however, we cannot exclude that this link may have been missed in some cases. For this reason, we are unable to exclude with certainty that transmission occurred within the Olimpico stadium. Notwithstanding, our data suggest that it is very unlikely that the stadium was a major transmission hotspot.

EURO2020 can provide elements to improve MGE epidemic preparedness planning. In particular, our data inform that targeted communication and control strategies to limit the impact of informal gatherings occurring outside official sites of MGEs should be further developed. In addition, our limits suggest that event-specific tracing studies or an enhanced surveillance would increase the robustness of retrospective epidemiological assessments of the impact of MGEs.
